# Macrophage migration inhibitory factor and placental malaria infection in an area characterized by unstable malaria transmission in central Sudan

**DOI:** 10.12688/f1000research.7061.1

**Published:** 2015-09-16

**Authors:** Reem Eltayeb, Naser Bilal, Awad-Elkareem Abass, Elhassan M. Elhassan, Ahmed Mohammed, Ishag Adam

**Affiliations:** 1 Faculty of Medical Laboratory Sciences, University of Khartoum, Khartoum, 11115, Sudan; 2 Faculty of Medicine, University of Geziera, Medani, 11111, Sudan; 3 Faculty of Medicine, Ribat University, Khartoum, 11111, Sudan; 4 Faculty of Medicine, University of Khartoum, Khartoum, 11115, Sudan

**Keywords:** Macrophage migration inhibitory factor (MIF), malaria, birth weight, hemoglobin, Sudan

## Abstract

**Background:** The pathogenesis of malaria during pregnancy is not fully understood. A proinflammatory cytokine, macrophage migration inhibitory factor (MIF) is suggested as a factor involved in the pathogenesis of malaria during pregnancy.

**Methods:** A cross-sectional study was conducted in Medani Hospital, Sudan to investigate MIF levels in placental malaria. Obstetrical and medical characteristics were gathered from each parturient woman using questionnaires. All women (151) were investigated for malaria using blood film and placental histology. MIF levels were measured using ELISA in paired maternal and cord blood samples.

**Results**: There were no *P. falciparum*-positive blood films obtained from maternal peripheral blood, placenta or cord samples. Out of 151 placentae, four (2.6%), one (0.7%), 32 (21.2%) showed acute, chronic and past infection on histopathology examinations respectively, while the rest (114; 75.5%) of them showed no signs of infection.There was no significant difference in the median (interquartile) of maternal [5.0 (3.7─8.8) vs 6.2(3.5─12.0) ng/ml, P=0.643] and cord [8.1(3.3─16.9) vs 8.3(4.2─16.9), ng/ml, P= 0.601] MIF levels between women with a positive result for placental malaria infection (n=37) and women with a negative result for placental malaria infection (n=114). In regression models placental malaria was not associated with maternal MIF, hemoglobin or birth weight. MIF was not associated with hemoglobin or birth weight **.**

**Conclusion**: There was no association between maternal and cord MIF levels, placental malaria, maternal hemoglobin and birth weight.

## Background

Malaria is a large public health problem in endemic tropical countries where there are over 30 million pregnancies at risk of malaria occur in Africa each year ^1^. Malaria during pregnancy can lead to adverse outcomes (both maternal and perinatal) e.g. anemia and low birth weight (LBW) ^2– 4^. Pregnant women in different regions of Sudan are susceptible to malaria, regardless of their age and parity ^5^. Malaria is associated with adverse pregnancy outcomes such as anemia ^6^ and LBW ^7^, and it is the main cause of maternal mortality ^8^.

The sequestration of *Plasmodium falciparum*–infected erythrocytes and accumulation of infected erythrocytes in placental intervillous spaces is responsible for the malaria-related pathologic changes in the placenta ^9, 10^. The exact mechanism by which malaria infection and placental inflammation result in fetal growth restriction and LBW is poorly understood. However, many chemokines and inflammatory cytokines are associated with malaria infection and malaria-related LBW ^11^.

Macrophage migration inhibitory factor (MIF) is a pro-inflammatory cytokine released from a variety of cells (T cells, monocytes, macrophages, blood dendritic cells, B cells, neutrophils, eosinophils, mast cells) and is implicated in the pathogenesis of sepsis, and inflammatory and autoimmune diseases ^12^. MIF has been observed in the human endometrium, placental villi, cytotrophoblasts, and it has been implicated in implantation and other reproductive functions ^13, 14^. Several studies have demonstrated the role of MIF in modulating malaria severity and pathogenesis ^15, 16^. It has recently been reported that women with a positive result for placental malaria had significantly higher intervillous plasma MIF levels than women with a negative result for placental malaria ^17^. There are few published data on MIF and placental malaria and none of them from Sudan. The current study was conducted in Medani Maternity Hospital, Central Sudan, to investigate MIF levels in women with placental malaria, and the effect – if any- on maternal hemoglobin and birth weight. This work is an addition to our previous research on malaria and its pathogenesis during pregnancy e.g. placental malaria infiltration ^18, 19^, hormones and cytokines ^20, 21^ complement, cytokines and malaria infections ^22, 23^.

## Material and methods

A cross-sectional study was conducted during the rainy and post-rainy season (September–November) 2013 in Medani Maternity Hospital, Central Sudan which is a referral tertiary hospital. Central Sudan is characterized by unstable malaria transmission and *P. falciparum* is the sole malaria parasite ^24^.

The sample size of 151 women was calculated to have 80% power and to detect a difference of 5% at α=0.05 and 10% of women might not respond or have incomplete data.

After signing an informed consent form, information on history of obstetrics, medical history, antennal attendance characteristics, and bed net use was gathered from participants using questionnaires applied by a trained medical officer. Maternal weight and height were measured and body mass index was calculated and expressed as weight (kg)/height (m) ^2^. Newborns were weighed immediately following birth using a Salter scale and the sex of each newborn was recorded.

### Giemsa-stained blood smears for light microscopy

5ml of blood (maternal and cord) were taken and allowed to clot and centrifuged for 10 minutes at 3000 rpm and the serum was separated and stored at -20°C till the analyses.

Maternal, placental, and cord blood films were prepared and stained using 10% Giemsa. If the slides were positive; the number of asexual parasites was counted per 200 leukocytes, assuming a leukocyte count of 8000 leukocytes/μl (for thick films) or per 1000 red blood cells (for thin films). Blood films were considered negative if no parasites were detected in 100 oil immersion fields of a thick blood film, which was double-checked in a blind manner by an expert microscopist. Maternal hemoglobin levels were measured by the HemoCue hemoglobinometer (HemoCue AB, Angelhom, Sweden) and recorded.

***Placental histology.*** The method used for placental histology was mentioned previously ^7, 18– 20^. In summary, a 3cm ^3^ placental sample was obtained from the maternal surface at a location approximately halfway between the umbilical cord and the edge of the placenta. Each biopsy sample was immediately placed in 10% neutral buffered formalin. The buffer was used to prevent formation of formalin pigment, which has similar optical characteristics and polarized light activity as malaria pigment ^25^. Placental biopsy samples were processed and were embedded in paraffin wax and 4mm thick slides were stained with hematoxylin-eosin and Giemsa. In these slides, placental malaria infection was characterized as follows ^26^: uninfected (no parasites or pigment), acute (parasites in intervillous spaces), chronic (parasites in maternal erythrocytes and pigment in fibrin, or cells within fibrin and/or chorionic villous syncytiotrophoblast or strom), and previous (no parasites, and pigment confined to fibrin or cells within fibrin).

### ELISA for measuring MIF levels

Maternal and cord serum levels were measured using a human MIF ELISA kit (BIOLEGEND catalogue number 438408, Pacific Heights Blvd, San Diego, USA) by following the manufacturer’s protocol.

### Statistical analysis

Data were entered into a computer using SPSS for windows (version 16.0). MIF data were not normally distributed and were compared between groups using Mann-Whitney U test. Multivariate analyses were performed using binary models for placental malaria infection as the dependent variable and linear models with hemoglobin, birth weight, and MIF (maternal and cord) levels as continuous dependent variables. Socio-demographic characteristics, education, antenatal care, residence, and placental malaria infections were the independent predictor of interest. Odds ratios (OR) and 95% confidence intervals (CI) were calculated and a P value of <0.05 was considered significant.

## Ethics

The study received ethical clearance from the Research Board at the Faculty of Medicine, University of Khartoum, Sudan.

## Results

The basic characteristics of the investigated women were shown in Table 1. There were no *P. falciparum*-positive blood films obtained from maternal peripheral blood, placenta or cord samples. Out of 151 placentae, four (2.6%), one (0.7%), 32 (21.2%) showed acute, chronic and past infection on histopathology examinations respectively, while the rest (114; 75.5%) of them showed no signs of infection.

**Table 1.** Basic characteristics of the pregnant women ( *n* = 151) included in the study at Medani Hospital, Sudan.

	Mean (SD)
Age (years)	26.9 (5.5)
Parity	2.0 (1.2)
Body mass index (weight (kg)/height (m) ^2^)	24.2 (2.3)
Hemoglobin, gm/dl	10.5 (1.1)
	Number (%) of
Primiparae	69 (45.6)
Lack of antenatal care	42 (27.8)
Education < secondary level	96 (63.5)
Rural residence	77 (50.9)
Uses bed nets	140 (92.7)
Has anemia	94 (62.2)

**Figure 1.**
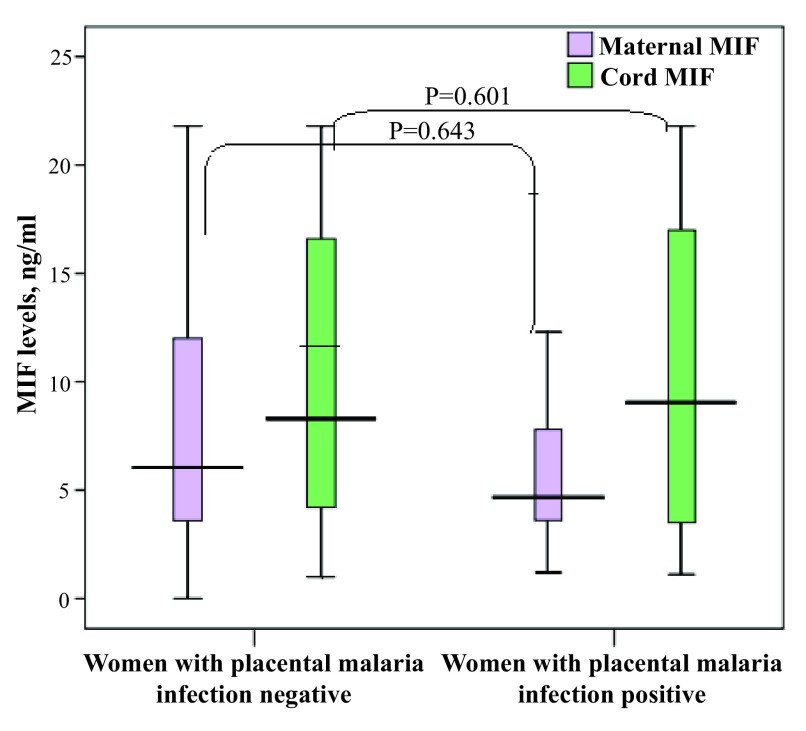
Maternal and umbilical cord macrophage migration inhibitory factor levels and placental malaria infection.

None of the investigated factors were associated with placental malaria infection, Table 2. There was no significant difference in the median (interquartile) of maternal [5.0 (3.7–8.8) vs 6.2(3.5–12.0) ng/ml, P=0.643] and cord [8.1(3.3–16.9) vs 8.3(4.2–16.9), ng/ml, P=0.601] MIF levels between women with a positive result for placental malaria infection (n=37) and women with a negative result for placental malaria infection (n=114; Figure 1).

**Table 2.** Factors associated with placental malaria among pregnant women at Medani Hospital, Sudan using univariate and multivariate analyses.

Variable	Univariate analyses			Multivariate analyses		
	OR	95% CI	P	OR	95% CI	P
Age, year	0.9	0.9–1.0	0.438	0.9	0.8–1.0	0.236
Parity	1.2	0.9–1.7	0.091	1.5	0.9–2.3	0.073
Gestational age, weeks	1.0	0.8–1.1	0.849	1.0	0.8–1.1	0.930
Lack of antenatal care	0.7	0.3–1.5	0.441	0.5	0.1–2.1	0.391
Rural residency	1.0	0.4–2.4	0.873	1.0	0.4–2.9	0.851
Education < secondary level	1.4	0.5–3.4	0.438	1.3	0.3–4.6	0.633
Using bed nets	4.2	0.8–20.1	0.151	6.6	1.1–37.7	0.032
Body mass index, weight (kg)/height (m) ^2^	1.0	0.8–1.1	0.927	0.9	0.8–1.2	0.909
Anemia	0.5	0.2–1.1	0.109	0.4	0.1–1.2	0.134
Macrophage inhibitory factor	0.3	0.1–1.1	0.094	0.9	0.9–1.0	0.904

OR = Odds ratio, CI = confidence interval

There was no significant difference in the median (interquartile) MIF levels [5.6(3.6–11.5) vs [7.3(3.0–9.7) ng/ml, P=0.516] between the maternal and cord samples.

In linear regression placental malaria was not associated with maternal MIF, hemoglobin or birth weight. Likewise MIF levels were not associated with maternal hemoglobin or newborn birth weight ( Table 3 and Table 4).

**Table 3.** Factors associated with maternal and cord MIF in pregnant women at Medani Hospital, Sudan using linear regression analyses.

Variable	Maternal MIF			Cord MIF		
	Coefficient	SE	P-value	Coefficient	SE	P-value
Age, year	0.209	0.112	0.065	–0.256	0.120	0.036
Parity	–0.596	0.480	0.217	0.394	0.511	0.442
Gestational age, weeks	–0.257	0.146	0.080	–0.105	0.156	0.503
Body mass index, (kg)/(m) ^2^	–0.526	0.233	0.025	0.373	0.251	0.140
Hemoglobin	0.579	0.468	0.218	0.142	0.498	0.776
Placental malaria infection	–0.236	1.261	0.852	–0.023	1.336	0.986
Macrophage inhibitory factor	–	–	–	0.473	0.093	< 0.001

MIF = macrophage inhibitory factor, SE = standard error

**Table 4.** Factors associated with maternal hemoglobin and birth weight levels at Medani Hospital, Sudan using linear regression analyses.

Variable	Hemoglobin			Birth weight		
	Coefficient	SE	P-value	Coefficient	SE	P-value
Age, year	0.028	0.022	0.202	0.010	0.010	0.335
Parity	–0.043	0.088	0.077	0.025	0.041	0.544
Gestational age, weeks	–0.044	0.028	0.112	0.011	0.012	0.367
Body mass index, (kg)/(m) ^2^	–0.029	0.045	0.524	0.021	0.020	0.307
Hemoglobin	–	–	–	0.022	0.040	0.583
Placental malaria infection	0.141	0.237	0.552	–0.122	0.106	0.253
Maternal MIF	0.018	0.018	0.316	0.002	0.008	0.847
Cord MIF	0.004	0.016	0.776	0.001	0.007	0.939

MIF = macrophage inhibitory factor, SE = standard error

There was a significant association between maternal blood/placental and cord MIF (0.473 ng/ml, P<0.001), Table 4.

Raw dataset for Eltayeb *et al.*, 2015 ‘Macrophage migration inhibitory factor and placental malaria infection in an area characterized by unstable malaria transmission in Central Sudan’ Basic characteristics of the women were gathered using questionnaires. Maternal and cord serum levels were measured using a human MIF ELISA kit. gestweeks=Gestational weeks; antenacare=had antinatal care (1=yes, 0=no); usebednet=used bed nets (1=yes, 0=no); useironfolic=used iron or folic acid (1=yes, 0=no); Wt=weight (Maternal;kg); Ht=height (Maternal;cm), Hb=haemoglobin (Maternal; g/dl); birthwt=birthweight (Child; kg); bloodgroup (Maternal): 0=A, 1=B, 2=AB 3=O; MIFcord= MIF levels in the umbilical cord (ng/ml); MIFmother (ng/ml). Raw data file openable by PSPP, available at https://www.gnu.org/software/pspp/. Click here for additional data file. Copyright: © 2015 Eltayeb R et al. 2015 Data associated with the article are available under the terms of the Creative Commons Zero "No rights reserved" data waiver (CC0 1.0 Public domain dedication).

## Discussion

The main findings of the current study were; there was no significant difference in the MIF levels in women positive for placental malaria infection and women negative placental malaria infection negative. There was no association between MIF, hemoglobin and birth weight.

This goes with previous reports where Singh *et al*. found no significant difference in the peripheral and cord MIF levels between women with placental malaria infections and women with placental malaria infections negative ^17^. It is worth mentioning that in Singh’s later study the MIF levels in the intervillous space (which we did not measure) were significantly higher than the peripheral and cord levels and higher in women with placental malaria infection compared with women negative for placental malaria infection ^17^. Furthermore, the observations of Singh *et al*. were based on microscopically-diagnosed placental malaria infection and in our cohort none of the women/placentae had microscopically detected malaria infections, except one that was diagnosed via histology. Yet high MIF was reported to be associated with adverse pregnancy outcome regardless of the presence of malaria infection ^17^. Likewise, Chaisavaneeyakorn *et al.* ^27^ observed high levels of MIF in the intervillous blood, compared with that in both peripheral and cord plasma and that intervillous (but not peripheral) MIF levels are associated with placental malaria among Kenyan women. Previous results obtained by Chaiyaroj and colleagues reported significantly higher MIF production by intervillous blood monocytes compared to peripheral ones and high MIF levels in placental plasma compared to paired peripheral plasma ^28^. Increased secretion of MIF by syncytiotrophoblasts was observed previously using an *in vitro* system ^29^. Generally MIF has been shown to play important roles during normal pregnancy ^30^, as well as in preterm delivery ^31^ and preeclampsia ^32^ and therefore, intervillous MIF would be expected to be high.

We have previously shown that immunomodulatory hormones (cortisol), cytokines, monocytes and macrophages were implicated in the pathogenesis of malaria during pregnancy which affected pregnant women regardless of their age and parity ^5, 7, 8, 18– 21^. Furthermore, histologic studies have shown that malaria-infected placentae have high numbers of macrophages loaded with malarial pigment and these cells could have a critical role in the clearance of the malaria parasites ^25^. Perhaps the high levels of MIF levels observed (by the later studies) in the placenta of women positive for malaria is induced by the malaria parasites that accumulated in the placenta, with MIF helping to retain macrophages in the placenta. Interestingly, it has been shown that MIF is effective in activating macrophages to clear/remove intracellular parasites e.g. *Leishmania major* ^33^.

As mentioned above, the malaria placental infections in the current study were past infections and this could explain the lack of significant difference in MIF levels. The other plausible explanation could be the submicroscopic/subpatent infections that we did not investigate in the current study. We have recently shown that in the same hospital, submicroscopic/subpatent infections that were detected by PCR rather than histology were significantly associated with low birth weight ^7^.

## Conclusion

The current study failed to show a significant association between maternal blood/placental and cord MIF levels, placental malaria, maternal hemoglobin or birth weight.

## Data availability

The data referenced by this article are under copyright with the following copyright statement: Copyright: © 2015 Eltayeb R et al.

Data associated with the article are available under the terms of the Creative Commons Zero "No rights reserved" data waiver (CC0 1.0 Public domain dedication). 

*F1000Research*: Dataset 1. Raw dataset for Eltayeb *et al.*, 2015 ‘Macrophage migration inhibitory factor and placental malaria infection in an area characterized by unstable malaria transmission in Central Sudan’, 10.5256/f1000research.7061.d102039 ^34^

## Supplementary materials

**Data collection questionnaire.**

Questionnaire was applied by a medical professional to participants in the study. ANC: antenatal care; HB: haemoglobin; WT: weight
