# Supplementary material for: Macrophage migration inhibitory factor and placental malaria infection in an area characterized by unstable malaria transmission in central Sudan
Source: F1000Res. 2015 Sep 16;4:824. [Version 1] doi: 10.12688/f1000research.7061.1 (PMC4863675; doi:10.12688/f1000research.7061.1)
Supplement: Supplementary file 2 [file f1000research-4-7601-s0001.tgz › c280be27-1669-416c-b69d-f1455cfd969a.docx]

**Flow chart of MIF at Medani Hospital, Sudan**

1. S number ------ name -------------------------------------------------------

2. Age ---------- parity--------------Gestational age-

3. Education Nil ----------Secondary-------- University and above---------

4. ANC Nil -----------1+2------- more than 2-----------

Use of bet Nets No Yes

5. Residence Urban----------Rural ----------

9. Blood group A ---- B------ AB--------- O--------

10. WT------ Height-------

11. HB

12**.** Maternal MIF Cord MIF

13. Birth weight
