# Supplementary material for: Macrophage migration inhibitory factor and placental malaria infection in an area characterized by unstable malaria transmission in central Sudan
Source: F1000Res. 2015 Sep 16;4:824. [Version 1] doi: 10.12688/f1000research.7061.1 (PMC4863675; doi:10.12688/f1000research.7061.1)
Supplement: Peer review history

# Peer review: Referee response for version 1

**DOI:** 10.5256/f1000research.7601.r13184

**Published:** 2016-05-04

**Authors:** Diana Boraschi

**Affiliations:** 1 Institute of Protein Biochemistry, National Research Council, Naples, Italy

I am in total favour of publishing reports of negative results, if the study is well conducted, as this one is. Malaria during pregnancy is an outstanding health issue that needs significant investigation. Biomarkers able to predict adverse outcomes are needed, and inflammation-related factors are a logical choice.

This study on the correlation between MIF (either in the maternal or in the cord blood), placental infection and pregnancy outcomes (maternal hemoglobin and birth weight) goes in this direction, and quite clearly shows that there is no correlation. The number of cases is possibly low, and no cases with blood malaria were found in the cohort. Although possibly preliminary, the study is nevertheless very clear and straight forward in its design, conduction and logical conclusions.

I have read this submission. I believe that I have an appropriate level of expertise to confirm that it is of an acceptable scientific standard.

# Peer review: Referee response for version 1

**DOI:** 10.5256/f1000research.7601.r10368

**Published:** 2015-09-22

**Authors:** Bernhard Zelger

**Affiliations:** 1 Department of Dermatology, Medical University Innsbruck, Innsbruck, Austria

Proper study, well outlined and performed.

Some minor critique regarding thickness of paraffin slides which should read 4 micromillimeter or with Greek letter μm, not "4mm". "strom" should read correctly "stroma". Finally, acronyms of authors in section "Authors contributions" are not clear to me with regard to the list of authors on title page. Please, amend, respectively.

I have read this submission. I believe that I have an appropriate level of expertise to confirm that it is of an acceptable scientific standard.
